# Forces maintaining the DNA double helix

**DOI:** 10.1007/s00249-020-01437-w

**Published:** 2020-05-27

**Authors:** Peter L. Privalov, Colyn Crane-Robinson

**Affiliations:** 1 Department of Biology, Johns Hopkins University, Baltimore, MD 21218 USA; 2 Biophysics Laboratories, School of Biology, University of Portsmouth, Portsmouth, PO1 2DT UK

**Keywords:** DNA, Stability, Hydrogen bonding, Base pair stacking, Hydration

## Abstract

Despite the common acceptance that the enthalpy of DNA duplex unfolding does not depend on temperature and is greater for the CG base pair held by three hydrogen bonds than for the AT base pair held by only two, direct calorimetric measurements have shown that the enthalpic and entropic contributions of both base pairs are temperature dependent and at all temperatures are greater for the AT than the CG pair. The temperature dependence results from hydration of the apolar surfaces of bases that become exposed upon duplex dissociation. The larger enthalpic and entropic contributions of the AT pair are caused by water fixed by this pair in the minor groove of DNA and released on duplex dissociation. Analysis of the experimental thermodynamic characteristics of unfolding/refolding DNA duplexes of various compositions shows that the enthalpy of base pairing is negligibly small, while the entropic contribution is considerable. Thus, DNA base pairing is entropy driven and is coupled to the enthalpy driven van der Waals base pair stacking. Each of these two processes is responsible for about half the Gibbs energy of duplex stabilization, but all the enthalpy, i.e., the total heat of melting, results from dissociation of the stacked base pairs. Both these processes tightly cooperate: while the pairing of conjugate bases is critical for recognition of complementary strands, stacking of the flat apolar surfaces of the base pairs reinforces the DNA duplex formed.

## Introduction

The understanding by Watson and Crick (1953) that two complementary strands of DNA are wound together into a double helix was a great discovery in biology, as it explained the mechanism of coding and replication of genetic information (Watson and Crick 1953). Moreover, it suggested that the physical basis of duplex stability is the hydrogen bonds between conjugate bases: two between A and T and three between C and G. This seemed to be confirmed by the optical observation that increase of CG content leads to a rise in DNA duplex thermostability (Marmur and Doty 1962). There were many subsequent attempts to estimate the thermodynamic contribution of base pairing to maintaining the double helix by measuring the heats of melting synthetic polynucleotides using conventional calorimetric instruments for liquids. These experiments seemed to confirm that the enthalpic contribution of both base pairs does not depend on temperature and is larger for the CG pair, as expected if the DNA double helix is maintained only by the hydrogen bonds between the bases [see, e.g., (Krakauer and Sturtevant 1968; Neuman and Ackerman 1969; Breslauer and Sturtevant 1977; Breslauer et al. 1986; Chalikian et al. 1999)]. It was expected, however, that a certain contribution to the DNA duplex formation might also result from the compactly packed flat base pairs (Sugimoto et al. 1996; SantaLucia 1998; Yakovchuk et al. 2006). Doubts concerning the experimental basis for all these suggestions stimulated the appearance of highly sensitive and precise calorimetric instruments designed for studying viscous and very dilute solutions: the Nano-DSC and Nano-ITC (Privalov 2012).

## Calorimetry of DNA duplexes

**Fig. 1:**
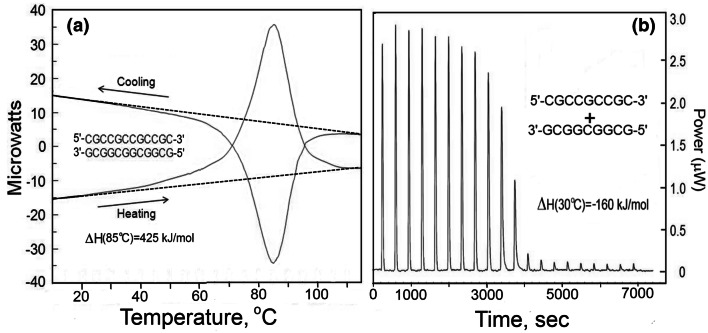
Original Nano-DSC recording of the heat effect on heating and subsequent cooling at a constant rate of 1 K/min of a 1 ml solution of 12 bp CG DNA duplex (left panel) and Nano-ITC titration of the 5′-CGCCGCCGCCGC-3′ strand into the 3′-GCGGCGGCGGCG-5′ complementary strand by injection of 10 µl portions into the 1 ml cell at 30 °C (right panel). Reproduced from Vaitiekunas et al. (2015)

Figure 1, left panel, illustrates a typical Nano-DSC recording of heating–cooling a 12 base pair all-CG duplex and the right panel illustrates a typical Nano-ITC recording of titrating one strand of the same DNA into its complementary strand, carried out at a constant temperature of 30 °C (Vaitiekunas et al. 2015).

**Fig. 2:**
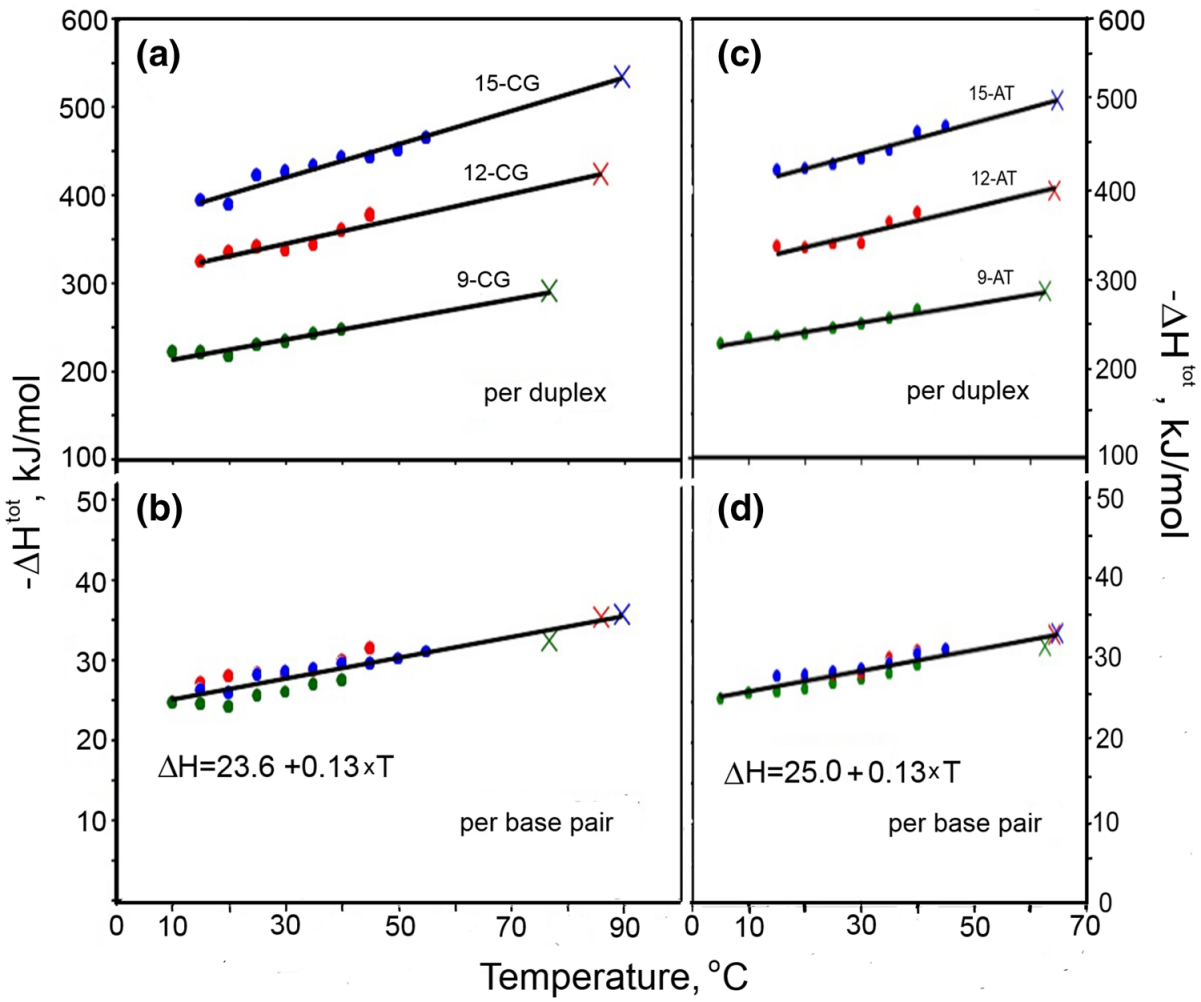
**a**, **c** The molar and **b**, **d** the specific molar (per base pair) enthalpies of formation, obtained from corrected ITC data, of three all-CG duplexes differing in the number of base pairs (left hand panels) and three AT-containing duplexes, each flanked with CGs (right hand panels): see Vaitiekunas et al. 2015. Crosses indicate the total enthalpies of forming the considered duplexes obtained from the DSC-measured excess heat of duplex melting and attributed to the transition temperatures, *T*_t_. All in 150 mM NaCl, 5 mM Na-phosphate, pH 7.4

The enthalpies derived from ITC experiments forming DNA duplexes of various size and composition at different fixed temperatures, after correcting for residual structure in the two strands, are plotted on the left side of each panel in Fig. 2. On the right hand side of each panel, crosses record the enthalpies of DSC experiments. It is seen that these two sets of thermodynamic data are in excellent agreement: the ITC data points extrapolate linearly exactly to the DSC-measured enthalpies. Analysis of these data (Vaitiekunas et al. 2015; Privalov and Crane-Robinson 2018a) led to two surprising conclusions: (a) the enthalpies of AT and CG unfolding are temperature dependent and increase with temperature rise, the heat capacity increment being similar for both base pairs: Δ*C*p = ∂Δ*H*/∂*T* = (0.13 ± 0.01) kJ/(K⋅mol-bp); (b) the enthalpic contribution of the AT base pair is larger than that of the CG base pair.

The last of these two conclusions is especially surprising since it was known that the presence of the AT base pair, which is held by two hydrogen bonds, lowers the stability of the DNA double helix relative to the three hydrogen bonded CG base pair that increases the stability.

**Fig. 3:**
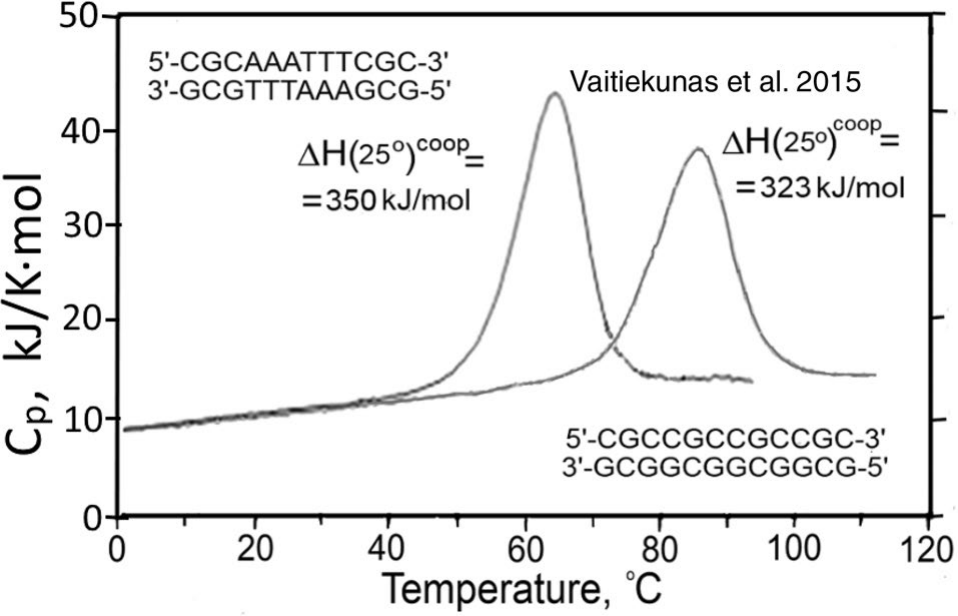
Comparison of the partial molar heat capacities of the 12 base pair all CG-duplex and the same length duplex having AT pairs in the central region. All measurements at the identical concentration of 283 µM in 150 mM NaCl, 5 mm Na-phosphate, pH 7.4. (See  Vaitiekunas et al. 2015 for more details)

Thus, although duplexes containing AT base pairs melt, as expected, at lower temperatures than those consisting only of CG pairs, absolutely unexpected was the finding that the less stable duplexes containing AT pairs melt with a higher heat effect (Fig. 3). Since the AT-containing duplex melts at a lower temperature than the same length all-CG duplex, one can conclude that: (c) the entropic contribution of the AT base pair also significantly exceeds that of the CG base pair, as does the enthalpy.

## The thermodynamic contributions of the base pairs

In contrast to the enthalpy of unfolding short DNA duplexes which, for all-CG duplexes, is exactly proportional to the number of base pairs, the entropy of duplex unfolding is not proportional to the number of the base pairs in the duplex because dissociation results in the appearance of a new kinetic unit, giving rise to the so-called translation entropy which should not depend on the DNA length.

The magnitude of the translational entropy was for a long time a matter of heated theoretical discussion: proposed values scattered from 230 J/(K mol) (Finkelstein and Janin 1989) to 400 J/(K mol) (Tidor and Karplus 1994). However, using a Nano-DSC it can be determined by measuring the unfolding entropies of all-CG DNA duplexes of various sizes and concentrations (Privalov and Crane-Robinson 2018b). It was found that the translation entropy increase on DNA duplex unfolding is perfectly described by the simple equation:

1 $$\Delta S^{{{\text{trans}}}} = R\ln \left( {\left[ {{\text{N}}_{{\text{o}}} } \right]/{2}} \right),$$

as originally predicted by Gurney (1953), but widely rejected as being physically inconsistent.

The other complication in analysis of the thermodynamic characteristics of DNA duplexes was the so-called near-neighbor effect: the DNA duplex stability dependence on the nature of its adjacent pair (Borer et al. 1974). However, using sufficiently long uninterrupted AT and CG sequences, this near-neighbor variation can be eliminated and the duplex unfolding enthalpies and entropies determined for AT and CG pairs with reasonable accuracy at standard temperature.

Table 1 gives the enthalpy, the conformational entropy, the Gibbs energy and also the heat capacity increment of AT and CG base pairs at the standard temperature of 25 °C under standard solvent conditions (150 mM NaCl, 5 mM Na-phosphate, pH 7.4).

**Table 1:** Contributions of the CG and AT base pairs to the enthalpy (Δ*H*), entropy (Δ*S*), Gibbs energy (Δ*G* = Δ*H* − *T*Δ*S*) and the heat capacity increment (ΔCp) on DNA duplex dissociation at 25 °C in 150 mM NaCl, 5 mM phosphate, pH 7.4, aqueous solution

Base pair	Δ*H*^coop^ (kJ/mol-bp)	Δ*S*^coop^ (J/(K mol-bp)	Δ*G*^coop^ (kJ/mol-bp)	Δ*C*_p_ [kJ/(K mol-bp)]
CG	19.0 ± 0.3	36.2 ± 0.2	8.2 ± 0.2	0.13 ± 0.01
AT	28.0 ± 0.3	73.5 ± 0.5	6.1 ± 0.2	0.13 ± 0.01

To minimize the near-neighbor effect, DNA duplexes containing at least 12 base pair of contiguous CG or AT sequences were chosen (Vaitiekunas et al. 2015). All data have been corrected for the translation entropy to exclude the effects of duplex concentration (Privalov and Crane-Robinson 2018b). These two precautions permitted a significant decrease in the error in estimating the thermodynamic contributions of the base pairs

## Analysis of the base pair contributions

It appears from Table 1 that the enthalpic and entropic contributions of the CG and AT base pairs at 25 °C are:

2 $$\begin{aligned} \Delta {\text{H}}^{{{\text{CG}}}} \left( {{25}\;^\circ {\text{C}}} \right) & = ({19}.0\; \pm \;0.{2}) \,{\text{kJ}}/{\text{mol-bp}}, \;{\text{and}} \\ \Delta {\text{H}}^{{{\text{AT}}}} \left( {{25}\;^\circ {\text{C}}} \right) & = ({28}.0\; \pm \;0.{3}) \,{\text{kJ}}/{\text{mol-bp}}, \\ \end{aligned}$$

3 $$\begin{aligned} \Delta S^{{{\text{CG}}}} \left( {{25}\;^\circ {\text{C}}} \right) & = ({36}.{2}\; \pm \;0.{2}) \,{\text{J}}/({\text{K}} \,{\text{mol-bp}}),\;{\text{and}} \\ \Delta S^{{{\text{AT}}}} \left( {{25}\;^\circ {\text{C}}} \right) & = ({73}.{5}\; \pm \;0.{5}) \,{\text{J}}/({\text{K}} \,{\text{mol-bp}}). \\ \end{aligned}$$

Comparison of these values shows that the duplex stabilizing effect of the CG base pair is larger than that of the AT, not because its enthalpic contribution is larger but because its entropic contribution is smaller (Vaitiekunas et al. 2015; Privalov and Crane-Robinson 2018b). The question is then: why are the entropic and also enthalpic contributions of the AT base pair larger than that for CG pairs?

## Role of water

One explanation for the observed difference between the enthalpies and entropies of the AT and CG base pairings is the water immobilized by the AT base pair in the minor groove of DNA (Drew and Dickerson 1981). Water molecules in the first hydration shell are fixed by the N3 of A and O_2_ of T bases and these are H-bonded to a second shell of waters, such that the oxygens of the first shell have the tetrahedral coordination seen in ice (Drew and Dickerson 1981; Kopka et al. 1983; Chiu et al. 1999). The second shell waters, however, are not as tightly bound as those in the first shell (Kopka et al. 1983). Release of this tightly bound minor groove water into the bulk solution will result in positive contributions to both the enthalpy and entropy of DNA melting. The contribution of these waters to the calorimetrically measured enthalpy and entropy of the AT pair can be approximated by the energetics of melting 1.5 mol of ice per AT pair, i.e., Δ*H* = 9 kJ/mol and Δ*S* = 33 J/(K.mol) for the enthalpic and entropic contributions. The net *intrinsic* enthalpic and entropic contributions are then:

4 $$\begin{aligned} \Delta {\text{H}}^{{{\text{CG}}}} \left( {{25}\;^\circ {\text{C}}} \right) & = ({19}.0\; \pm \;0.{2}) \,{\text{kJ}}/{\text{mol-bp}}\;{\text{and}} \\ \Delta {\text{H}}^{{{\text{AT}}}} \left( {{25}\;^\circ {\text{C}}} \right) & = ({19}.0\; \pm \;0.{3}) \,{\text{kJ}}/{\text{mol-bp}}, \\ \end{aligned}$$

5 $$\begin{aligned} \Delta {\text{S}}^{{{\text{CG}}}} \left( {{25}\;^\circ {\text{C}}} \right) & = ({36}.{2}\; \pm \;0.{2}) \,{\text{J}}/({\text{K}} \,{\text{mol-bp}})\;{\text{and}} \\ \Delta {\text{S}}^{{{\text{AT}}}} \left( {{25} \;^\circ {\text{C}}} \right) & = ({4}0.{5}\; \pm \;0.{2}) \,{\text{J}}/({\text{K}} {\,\text{mol-bp}}). \\ \end{aligned}$$

It is immediately apparent that whilst the intrinsic entropy of melting an AT pair is greater than that of a CG pair by about 4 J/K mol-bp, the enthalpies are essentially the same.

## Forces holding the DNA base pairs

If it is assumed that the DNA double helix is maintained only by the hydrogen bonds between conjugate bases, then dividing the enthalpy, entropy and the Gibbs energy values of the CG base pair (Table 1) by the number of hydrogen bonds between these bases, one finds that a single hydrogen bond should contribute about 6.3 kJ/mole-bp in enthalpy and 12 J/(K mole-bp) in entropy. These values very substantially exceed those to be expected for the breakage of a single hydrogen bond between polar groups in aqueous media where disrupted hydrogen bonds between the groups of proteins or nucleic acids switch immediately to the surrounding water molecules. The overall enthalpy of such a process should be quite small, while the entropy is expected to be strongly negative because water molecules become ordered around the newly exposed polar groups (Makhatadze and Privalov 1995).

Comparison of the enthalpies of the CG base pair with the corrected AT base pair—given in Eqs. (4) and (5)—shows an essential equivalence. Since the CG base pair is held by three hydrogen bonds, while the AT by two, one can therefore conclude that the enthalpic component of base pair hydrogen bonding is indeed negligibly small. Comparison of the entropic contribution of the CG and AT base pairs shows that it is negative and amounts to about − 4.0 J/K mol per bond. It thus appears that at the standard temperature 25 °C = 298 K, a single hydrogen bond provides about Δ*S* × *T* = 4.0 J/(K mol-bp) × 298 K = 1.2 kJ/mol to the Gibbs energy of base pairing. It follows then that the Gibbs energy of a single CG base pair (held by 3 hydrogen bonds) amounts to 3.6 kJ/mol, while for a single AT base pair (held by two hydrogen bonds) it amounts to 2.4 kJ/mol. In contrast, however, these two base pairs provide essentially nothing to the enthalpy of duplex stabilization. This immediately raises the question: what then is the source of the calorimetrically observed large heat effect of DNA duplex melting, i.e., the source of the enthalpy of DNA duplex dissociation?

## The enthalpy of DNA unfolding

The calorimetrically observed large enthalpy of DNA melting results partly from release of the water fixed in the minor groove of DNA, but also from unpacking the stacked flat base pairs in the DNA duplex. The first provides about 9 kJ per mole of AT base pair, but the remainder of the enthalpy can result only from melting the stacked apolar base pairs. The unpacking of stacked base pairs is responsible not only for the large magnitude of the DNA melting enthalpy, but also for its dependence on temperature, that is, for the heat capacity increment specific for DNA unfolding (Fig. 2; Table 1).

The experimentally observed heat capacity increment on DNA duplex dissociation has been the subject of controversy in DNA energetics. It is known that hydration of polar groups results in a partial heat capacity *decrement*, in contrast to apolar group hydration that results in a partial heat capacity* increment* (Makhatadze and Privalov 1995; Privalov and Gill 1988; Privalov and Makhatadze 1992; Spolar et al. 1992). According to Makhatadze and Privalov (1995), the heat capacity effect of hydration of the apolar and polar groups is expressed by the equation:

6 $$\Delta {\text{Cp}}\,\left( {{25}\;^\circ {\text{C}}} \right) = {2}.{14}^{.} \Delta {\text{ASA}}_{{{\text{apolar}}}} - {1}.{27}^{.} \Delta {\text{ASA}}_{{{\text{polar}}}} .$$

Thus, breaking the hydrogen bonding between the paired bases of DNA, which results in exposure of new highly polar groups, should proceed with a partial heat capacity decrement. In fact, as demonstrated in Fig. 2, the enthalpy of DNA unfolding increases with temperature rise, i.e., DNA unfolding results in a partial heat capacity increase! The clear heat capacity increment on duplex dissociation shows that this process proceeds not only with breaking the hydrogen bonds between polar groups and exposure of these groups to water, but also with breakage of the contacts between the apolar bases tightly packed in the DNA interior and exposure of their apolar groups to water, i.e., their hydration.

## Surfaces exposed upon DNA duplex dissociation

There are now many well-defined crystallographic structures of DNA duplexes that permit estimation of their exposed surfaces (Woods et al. 2004; Narayana and Weiss 2009; Garcia et al. 2019). Unfortunately, we do not have the structure of an unfolded DNA since its separated strands, being highly flexible, are in extensive thermal motion. Nevertheless, modeling unfolded DNA by its isolated disordered single strands, one can determine by the Naccess program the approximate increase in water-accessible surface areas (ASA) of the polar and non-polar groups on separation of the DNA strands (Dragan et al. 2019). Such an analysis for several DNA duplexes showed that their unfolding indeed results in significant increases in the exposed apolar and polar surfaces. Increase of the polar surfaces upon DNA unfolding includes a large component from disruption of the hydrogen bonding between the paired polar groups of the bases, while increase of the apolar surfaces results largely from dissociation of the stacked bases to expose their apolar surfaces to water. It appears that in unfolded DNA, the newly exposed polar and apolar surfaces are similar in area. However, since the intrinsic heat capacity increase from apolar surface hydration (i.e., per Å^2^ of ΔASA) substantially exceeds the decrease from polar group hydration (Eq. 6), the overall heat capacity effect of DNA duplex unfolding is positive: the average expected heat capacity effect of both AT and CG pair dissociation appears to be Δ*C*p = (0.14 ± 0.03) kJ/(K mol-bp). This value is surprisingly close to the calorimetrically measured heat capacity increment on DNA duplex unfolding, (0.13 ± 0.01) kJ/(K mol-bp) (see Fig. 2). The close correspondence of the measured and calculated heat capacity effects shows that the approximation used for modeling the unfolded duplex as its isolated single strands (Dragan et al. 2019) is valid. This agreement also shows that upon strand dissociation not only the polar groups involved in hydrogen bonding of the conjugate bases become exposed to water, but also the apolar surfaces of those bases.

It should be noted that when recalculated per gram, the specific heat capacity increment on DNA duplex unfolding is significantly smaller than the specific heat capacity increment on the unfolding of globular proteins (Privalov 2012; Privalov and Makhatadze 1992). This shows that the concentration of apolar groups in the DNA interior is significantly lower than in globular proteins. The high concentration of apolar groups in proteins is precisely what makes them globular. It follows, therefore, that the contacts between the apolar bases in the DNA double helix are sufficient only for its linear organization.

## Contribution of the bases to the DNA duplex stability

Earlier studies of the stability of the DNA duplex were strongly affected by the Watson and Crick model of DNA and by the first optical observations that the presence of three hydrogen-bonded CG base pair increases the DNA duplex stability. These encouraged the belief that H bonding is the primary determinant of duplex stability, i.e., it represents its physical basis. However, the hydrogen bonds, which are entropic, cannot be responsible for the large enthalpy of DNA unfolding and certainly not for the dependence of this enthalpy on temperature, i.e., for the heat capacity increment specific for DNA melting. It follows therefore that there must be another source of the enthalpy and this can only be the tightly packed apolar surfaces of the base pairs in the DNA duplex. Disruption of the extended van der Waals contacts between the flat apolar surfaces of the bases will require considerable enthalpy and the consequent exposure of their apolar surfaces to water results in a significant heat capacity increment. It is this heat effect and the heat capacity increment that are calorimetrically recorded upon DNA melting (see Fig. 2). The question is then: how much Gibbs energy of stabilization is provided by the pairing of conjugate bases and by the stacking of their flat surfaces?

As shown in “Analysis of the base pair contributions”, the Gibbs energy of the CG base pair, held by three hydrogen bonds, is entirely entropic and amounts to 3.6 kJ/mol-bp, while the overall Gibbs energy of this base pair is 8.9 kJ/mol-bp (Table 1). The difference between these two values, about 5.3 kJ/mol-bp, can be provided only by the stacked bases. It appears therefore that base stacking is responsible for about 60% of the overall Gibbs energy of the CG base pair. Similarly, the two hydrogen bonds of the AT base pair provide only 2.4 kJ/mol-bp to the Gibbs energy, while the overall Gibbs energy of this base pair is 6.1 kJ/mol-bp (Table 1). It follows that in the case of AT base pairs, stacking is responsible for 61% of the total Gibbs energy. It is notable, however, that for both AT and the CG pairs, the stacking of bases is responsible for essentially all the enthalpy of DNA melting!

Calorimetric study of the DNA duplex has thus confirmed the earlier expectations that significant contributions to DNA duplex stability result from the compact packing of the flat bases (Sugimoto et al. 1996; SantaLucia 1998; Yakovchuk et al. 2006). Moreover, calorimetry has shown that the contributions of base stacking and base pairing to the Gibbs free energy of duplex stabilization are of a similar order but have different thermodynamic origins.

## Analogy between the DNA double helix and the *α-*helix

From the initial discovery of the α-helical conformation by Pauling (Pauling et al. 1951), it was held that hydrogen bonding between the *i*th and (*i* + 4)th residues along the polypeptide chain is primarily responsible for its stability. Calorimetric studies of α-helix melting showed, however, that it proceeds with considerable heat absorption, i.e., a large enthalpy amounting to (66 ± 2) kJ/mol-residue and a heat capacity increment amounting to (0.46 ± 0.10) kJ/(K mol-residue) (Privalov 2012; Taylor et al. 1999). Thus, this enthalpy substantially exceeds that expected for the breakage of a single hydrogen bond between amino acid residues in aqueous solution, while the heat capacity increment shows that α-helix unfolding results in exposure of considerable apolar surface. The question is then: what might be the source of this unexpectedly large and temperature-dependent enthalpy of α-helix melting in the presence of water? This becomes evident from the crystallographically resolved interior of the α-helix showing that this is not empty, but contains a tightly packed core formed from the apolar groups of the constituent amino acids (Privalov 2012). One can thus expect that the positive enthalpy of α-helix melting in aqueous solution results mainly from melting this apolar core. It follows that this apolar core in the α-helix plays the same role as the stacked flat apolar surfaces of the base pairs in the case of the DNA double helix in promoting the linearity of the structure. Indeed, it was found that the π-helix, which is less compact than the α-helix and does not form a compact apolar core, is much less stable than the α-helix (Privalov 2012).

## Conclusion

It appears that base pairing and base stacking are two tightly interconnected processes in DNA folding. Indeed, pairing of bases requires their proper orientation, while the proper orientation of the base pair surfaces leads to their stacking. Vice versa, base pair stacking assumes the proper orientation of the conjugate polar groups of the bases that is required for their hydrogen bonding. Thus, these two steps represent a single cooperative act of DNA double helix formation. The cooperation of entropy-driven base pairing with enthalpy-driven base stacking explains the extreme efficiency of DNA double helix propagation that proceeds with an increase in its rigidity. In this cooperative folding process, while the hydrogen bonds between the conjugate bases are of critical importance for the proper alignment of the two complementary strands of DNA, the double helix formed is reinforced by the simultaneous stacking of the flat apolar surfaces of the base pairs.
